# A New Approach for Clustered MCs Classification with Sparse Features Learning and TWSVM

**DOI:** 10.1155/2014/970287

**Published:** 2014-02-09

**Authors:** Xin-Sheng Zhang

**Affiliations:** School of Management, Xi'an University of Architecture and Technology, Xi'an, Shaanxi 710055, China

## Abstract

In digital mammograms, an early sign of breast cancer is the existence of microcalcification clusters (MCs), which is very important to the early breast cancer detection. In this paper, a new approach is proposed to classify and detect MCs. We formulate this classification problem as sparse feature learning based classification on behalf of the test samples with a set of training samples, which are also known as a “vocabulary” of visual parts. A visual information-rich vocabulary of training samples is manually built up from a set of samples, which include MCs parts and no-MCs parts. With the prior ground truth of MCs in mammograms, the sparse feature learning is acquired by the *l*_*P*_-regularized least square approach with the interior-point method. Then we designed the sparse feature learning based MCs classification algorithm using twin support vector machines (TWSVMs). To investigate its performance, the proposed method is applied to DDSM datasets and compared with support vector machines (SVMs) with the same dataset. Experiments have shown that performance of the proposed method is more efficient or better than the state-of-art methods.

## 1. Introduction

Breast cancer is the most common tumor disease in women, with the increasing incidences in recent years. And also, it is one of the major death causes among middle-aged women in the world. Currently, digital mammograms are one of the most reliable methods to perform the early diagnosis, which is very important for the effectiveness of treatment methods.

In digital mammograms, an important sign of the early breast cancer is the existence of MCs. They always exist in 30%–50% of mammographically diagnosed cases, which are present with tiny bright spots of different morphology. Microcalcifications are small calcifications with different shapes and densities, approximately 0.1–1 mm in diameter. Isolated microcalcifications are not dangerous, but a microcalcification cluster might be an early sign of breast cancer [1], which is a region including more than three microcalcifications per 5 mm × 5 mm.

However, there is only about 3% of useful information in mammograms, which can be seen by doctors with the naked eye. Due to the fact that most details in mammograms cannot be perceived by human eyes, it is even very difficult for a skillful radiologist to find the sign of early breast cancer, that is, MCs, as a result missing the best time for treatment. So, one of the key techniques for early diagnosis of the breast cancer is to detect MCs and to judge whether they are malignant or not in mammograms.

**Figure 1:**
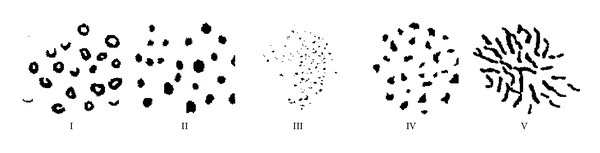
Le Gal's MCs classification standards. Type I: annular; Type II: regularly punctiform; Type III: dusty; Type IV: irregularly punctiform; Type V: vermicular calcification.

According to recent researches, there are several existing criteria to characterize the MCs shape properties. Among them, one of the well known is the category criterion proposed by Le Gal et al. [2], which illustrates five groups (shown in Figure 1). Different groups identify different kinds of MCs in the ascending order of the degree of malignancy. As shown in Figure 1, the first group describes the O-shaped calcifications and partially calcified ones, known as teacup calcifications. The second one includes regular and round calcifications with uniform density. The third is composed of calcifications with the same shape and smaller size than the second one. Class IV is also called salt shaped, which is irregular MCs related to the high degree of malignancy. Type V is also closely related to a very high degree of malignancy, which is called vermicular shaped.

Up till now, computer aided diagnosis (CAD) is still a useful tool in breast cancer detection to improve the accuracy of radiologists and to help radiologists to read mammogram films. It may provide good help to radiologists in interpreting mammograms to detect MCs and classify them into malignancy or not. A large number of researchers in this field have been trying to find effective methods to automatically detect MCs and categorize them as normal, benign, or malignant.

Because it is very important in breast cancer diagnosis, the detection accuracy of MCs has become a crucial application task and research. Recently a lot of methods have been developed. These approaches have been also greatly assisting radiologists and doctors in diagnosing the disease [3–5]. Among them, several methods focus on image enhancement and segmentation of regions of interests (ROIs), such as local threshold and classical image filter [6, 7], optimal filters [7, 8], fractal models [9], wavelet and multiscale analysis [6, 10, 11], and mathematical morphology [12, 13]. Various classification approaches based on machine learning have also been presented to detect and classify MCs, such as rule-based systems [14], fuzzy logic systems [15–19], statistical methods based on Markov random fields [20, 21], support vector machines (SVMs) [20, 22], twin support vector machines (TWSVMs) [23, 24], and twin support tensor machines (TWSTMs) [24]. In the last ten years, a lot of researches reported in the literatures have used neural networks for MCs characterization [9, 10, 12, 25–28]. With the development of SVMs, various SVMs have been designed to categorize ROIs [29].

However, how to successfully apply the mammography technology to detect breast cancer and design a breast cancer detection system greatly depends on the careful designing of the two important modules: feature selection and sample classification. A lot of well-established methods have been proposed to address this challenge problem. According to [30], these methods can be categorized into the following groups: (a) traditional methods, such as linear discriminant analysis (LDA), K-nearest neighbor (KNN), logistic regression (LR), and generalized partial least square (GPLS); (b) classification trees and aggregation methods, such as classification and regression tree (CART), bagging and boosting (BB), random forest (RF), and ensemble learning (EL); (c) machine learning based methods, such as neural network (NN), support vector machines (SVMs), and twin support vector machines (TWSVMs); and (d) generalized methods, such as flexible discriminant analysis (FDA), bias discriminant analysis (BDA), mixture discriminant analysis (MDA), and shrunken centroid method.

To detect the early sign of this disease and to aid doctors to diagnose breast cancer in early stage, a novel approach for MCs classification is proposed based on sparse feature learning and representation with TWSVMs, which is inspired by the recent progress in *l*_1_-norm minimization-based approaches [31, 32]. These approaches, such as compressive sensing for sparse signal reconstruction, basis pursuit denoising, and the Lesso algorithm for features selection, have been well developed. Inspired from the above well-established approaches, our approach presented in this paper is based on the belief that the key problem to finding a solution to the problem depends on learning the suitable representation.

Especially, to extract high-level and conceptual information, for example, the existence of an MC in a mammogram block, it is very important for us to convert the low-level input, such as the pixel value, to high-level and more meaningful representations. Through this transformation, the feature learning and detection process will be well constructed.

Ideally, a test example can be represented just from the training samples of the same category. Therefore, when the test sample is expressed as a linear combination of the entire training sample, the coefficient vector will be sparse. That is, there will be relatively few nonzero coefficients in the vector. Test samples from the same category will have a similar sparse representation, while test samples from different categories will lead to different sparse representations. So the sparse representation coefficients can be treated as the more meaningful and discriminant information for the samples classification. In order to get the sparse coefficient vector, we use *l*_1_-regularized least square [33] to solve the problem.

To achieve a good performance for MCs detection, we designed two methods to achieve the goal of the detection system. The first one is the sparse discriminant analysis algorithm, which is achieved by computing the residuals of sparse coefficients of the test sample between the centroid sparse coefficients of training samples. As we have known traditional supervised learning methods always use a training procedure to create a classification model for testing. But the proposed sparse representation based approach does not contain the separate training and testing sections. We directly achieved the classification goal out of the testing samples' sparse representation according to the training samples. Another unique feature of the new method is that no model selection is needed. The second one is designed by the combination of sparse representation and the state-of-the-art classifier TWSVMs. We employ the sparse representation approach as a feature learning method in terms of the coefficient vector for samples feature extraction, and then we feed it with the trained TWSVMs to formulate the detection method as a supervised learning approach.

The paper is organized in five sections. Technology backgrounds of our approach are presented in Section 2; sparse representation based MCs detection algorithm and TWSVM based MCs detection with sparse representation are given in Section 3. Thereafter, the sparse representation based MCs detection algorithms are formulated, and experimental results are illustrated in Section 4 accordingly. Finally, conclusions are drawn in Section 5.

## 2. Technology Backgrounds

### 2.1. Image Sparse Representation and Learning

Given a training dataset {(**x**_*i*_, *l*_*i*_); *i* = 1,…, *n*}, **x**_*i*_ ∈ *R* ^*d*^, *l*_*i*_ ∈ {1,2,…, *N*}, where **x**_*i*_ is the *i*th sample, a *d*-dimension column vector contains MCs features, *d* is the number of features, and *l*_*i*_ represents the label of the *i*th sample with *N* as the number of categories, and a test sample **y** ∈ *R* ^*d*^, the problem of sparse representation aims to find a column vector **c** = [*c*_1_, *c*_2_,…, *c*_*n*_]^*T*^ such that

(1) $$\begin{array}{c} y=c_{1}x_{1}+c_{2}x_{2}+\cdots +c_{n}x_{n}, \end{array}$$

and ||*c*||_0_ is minimized, where ||*c*||_0_ represents the *l*_0_-norm, which means that it is equal to the number of non-zero components in the vector **c**.

Suppose that we define a matrix by putting **x**_*i*_ as the *i*th column of **A** = [**x**_1_, **x**_2_,…, **x**_*n*_]; we can convert the problem of sparse representation into

(2) $$\begin{array}{c} c=\underset{c^{\prime }\in R^{n}}{\min}{||c^{\prime }||}_{0}\;\text{subject}\;\text{to}\;\;y=Ac. \end{array}$$

How to get the close solution of the sparse representation problem is NP-hard, because it is a combinational optimization. If we replace the *l*_0_-norm in (2) with *l*_*p*_-norm, an approximation solution can be gotten. Thus,

(3) $$\begin{array}{c} c=\underset{c^{\prime }\in R^{n}}{\min}{||c^{\prime }||}_{p}\;\text{subject}\;\text{to}\;\;y=Ac, \end{array}$$

where the *l*_*p*_-norm of a vector **u** is defined as ||**u**||_*p*_ = (∑_*i*_|**u**_*i*_|^*p*^)^1/*p*^. A generalized version of (3), which allows for certain degree of noise, is defined to find a vector **c**, when the following objective function is minimized:

(4) $$\begin{array}{c} J\left(c,\lambda \right)=\underset{c}{\min}\left\{{||Ac-y||}_{2}+\lambda {||c||}_{p}\right\}, \end{array}$$

where the scalar regularization *λ* is a positive parameter, which balances the trade-off between sparsity and reconstruction error.

Recently, development in the theory of compressed sensing and sparse representation reveals that, if the solution of (2) is sparse enough, the solution of the *l*_0_-minimization problem is equal to the solution of the following *l*_1_-minimization problem [33], which takes *p* = 1 in (4):

(5) $$\begin{array}{c} c=\underset{c^{\prime }\in R^{n}}{\min}{||c^{\prime }||}_{1}\;\text{subject}\;\text{to}\;\;y=Ac,\\ J\left(c,\lambda \right)=\underset{c}{\min}\left\{{||Ac-y||}_{2}+\lambda {||c||}_{1}\right\}. \end{array}$$

We can solve the problem in polynomial time by quadratic programming or standard linear programming methods. If the solution is very sparse, there will be more efficient methods to solve this problem.

### 2.2. Twin Support Vector Machines

Twin support vector machines (TWSVMs) are a new binary data classifier proposed by Jayadeva et al. [34], which aims at obtaining two nonparallel planes close to two nonparallel planes such that each plane is closer to one of the two classes and is as far as possible from the other. There are two quadratic programming problems (QPPs) to be solved in the TWSVMs. But each QPP is of a smaller size instead of a large one as we have in the traditional SVMs. So, to some extent, TWSVMs work much faster than the stand SVMs facing the same classification problem; that is, it is more efficient

According to (6) we can solve the following two QPPs to obtain the TWSVM classifier:

(TWSVM1)

(6)  min⁡w(1),b(1),q12(Aw(1)+e1b(1))T(Aw(1)+e1b(1))+c1e2Tqs.t. −(Bw(1)+e2b(1))+q≥e2, q≥0,

(TWSVM2)

(7) min⁡w(2),b(2),q 12(Bw(2)+e2b(2))T(Bw(2)+e2b(2))+c2e1Tqs.t. (Aw(2)+e1b(2))+q≥e1, q≥0,

where **e**_1_ and **e**_2_ are vectors of ones with proper dimensions and *c*_1_, *c*_2_ > 0  are scalar parameters.

The first term, in the objective function of (6) or (7), is defined by the sum of squared distances from the points of each class to the hyper plane. So, minimizing the objective function tends to keep the hyper plane close to points which belong to one class (say class +1). Meanwhile, the constraints request of the hyper plane to be a distance of no less than 1 to the points belonging to the other class (say class −1). In the model, we use a set of error variables to measure the computing error, whenever the distance to the hyper plane is less than 1. In (7), the objective function aims at minimizing the total error variables, so as to attempt to minimize the misclassification with respect to the points belonging to class −1.

TWSVMs are composed of two QPPs. And the objective function in each QPP, corresponding to a particular class and constrains, is determined by the patterns belonging to the other class. In TWSVM1, patterns of class +1 are clustered near the hyper plane **x** ^*T*^ **w** ^(1)^ + *b* ^(1)^ = 0. Similarly, in TWSVM2, patterns of class −1 are clustered around the hyper plane **x** ^*T*^ **w** ^(2)^ + *b* ^(2)^ = 0. The Lagrange equation corresponding to TWSVM1 is given by

(8) L(w(1),b(1),q,α,β) =12(Aw(1)+e1b(1))T(Aw(1)+e1b(1))+c1e2Tq  −αT(−(Bw(1)+e2b(1))+q−e2)−βTq,

where **α** = (*α*_1_,*α*_2_,…,*α*_*m*_2__)^*T*^ and **β** = (*β*_1_,*β*_2_,…,*β*_*m*_2__)^*T*^ are the vectors of Lagrange multipliers. The Karush-Kuhn-Tucker (K.K.T.) conditions for TWSVM1 are given by

(9) $$\begin{array}{c} A^{T}\left(Aw^{\left(1\right)}+e^{1}b^{\left(1\right)}\right)+B^{T}\alpha =0,\\ {e_{1}}^{T}\left(Aw^{\left(1\right)}+e^{1}b^{\left(1\right)}\right)+e_{2}^{T}\alpha =0,\\ c_{1}e_{2}-\alpha -\beta =0,\\ -\left(Bw^{\left(1\right)}+e_{2}b^{\left(1\right)}\right)+q\geq e_{2},\;q\geq 0,\\ \alpha^{T}\left(-\left(Bw^{\left(1\right)}+e_{2}b^{\left(1\right)}\right)+q-e_{2}\right)=0,\;\beta^{T}q=0,\\ \alpha \geq 0,\;\beta \geq 0. \end{array}$$

If we define $H=\left[\begin{array}{cc} A & e_{1} \end{array}\right]$, $G=\left[\begin{array}{cc} B & e_{2} \end{array}\right]$, and **u** = [**w** ^(1)^;  *b* ^(1)^]^*T*^, we can get

(10) $$\begin{array}{c} u=-{\left(H^{T}H\right)}^{-1}G^{T}\alpha , \end{array}$$

where **H** ^*T*^ **H** is positive semidefinite. With the K.K.T. conditions and the Lagrange equation of problem TWSVM1, we can get the Wolfe dual of TWSVM1 as follows:

(DTWSVM1)

(11)   max⁡α e2Tα−12αTG(HTH)−1GTαs.t. 0≤α≤c1.

Similarly, if we consider TWSVM2; then we can also obtain its dual as

(DTWSVM2)

(12)   max⁡γ e2Tγ−12γTH(GTG)−1HTγs.t. 0≤α≤c1.

And we define the augmented **v** = [**w** ^(2)^;  *b* ^(2)^]^*T*^ as

(13) $$\begin{array}{c} v=-{\left(G^{T}G\right)}^{-1}H^{T}\gamma . \end{array}$$

If we get the vector **u** and **v** from (10) and (13), the hyper planes

(14) $$\begin{array}{c} x^{T}w^{\left(1\right)}+b^{\left(1\right)}=0,\;\;x^{T}w^{\left(2\right)}+b^{\left(2\right)}=0 \end{array}$$

can be obtained. Suppose that we have a new sample **x** ∈ *R* ^*n*^, which is assigned to class *p*  (*p* = 1,2). The data belongs to which category will be determined by the closer plane to the sample; that is,

(15) $$\begin{array}{c} x^{T}w^{\left(p\right)}+b^{\left(p\right)}=\underset{l=1,2}{\min}\left|x^{T}w^{\left(l\right)}+b^{\left(l\right)}\right|, \end{array}$$

where |·| represents a vertical distance of the point **x** to the plane **x** ^*T*^ **w** ^(*l*)^ + *b* ^(*l*)^ = 0, *l* = 1,2.

**Figure 2:**
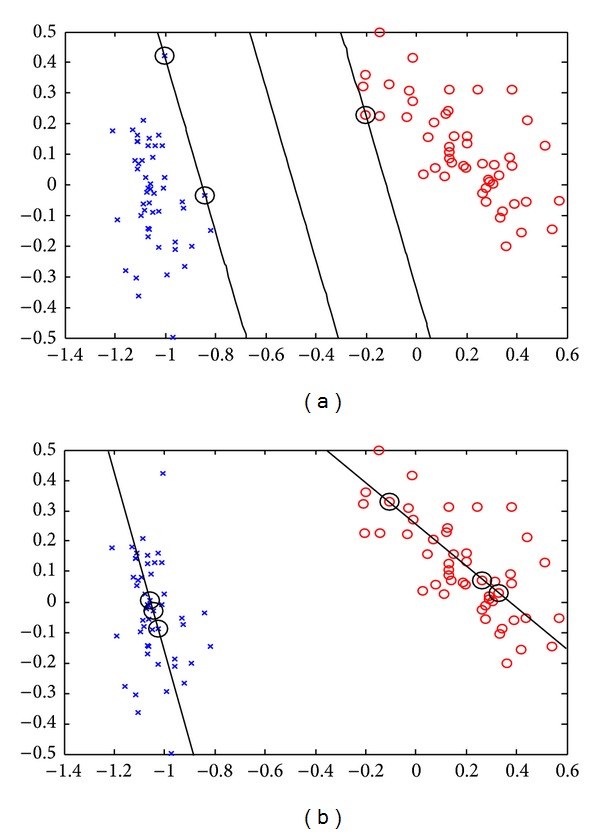
An example of SVMs and TWSVMs: (a) SVMs and (b) TWSVMs. All the points of class +1 are represented by a “×” and those of class −1 by “○.”

From the K.K.T. conditions, we can observe that patterns lie on the hyper plane given by **x** ^*T*^ **w** ^(1)^ + *b* ^(1)^ = 0  of class −1 given 0 < *α*_*i*_ < *c*_1_ (*i* = 1,2,…, *m*_2_), and such patterns of class −1 are called support vectors of class 1 according to class −1 as they play a key role when we determine the required hyper plane. A similar observation can be gotten from the problem TWSVM2. From the example shown in Figure 2, one can find the difference between TWSVMs and traditional SVMs.

## 3. **Materials and Methods**

### 3.1. Database and Evaluation Metrics

The digital database for screening mammography (DDSM) [35] was built by the University of South Florida, which is available for research at [36]. In our experiments, all images manually selected from this database are intensity images, digitized at 43.5 *μ*m/pixel and 12-bit gray scale. To evaluate our methods, we totally selected a set of 267 images from the DDSM, which are all clinical mammograms, to form an evaluation database.

To summarize quantitatively the performance of the proposed method, we used receiver operating characteristic (ROC) curves [37]. Receiver operating characteristic analysis is based on statistical decision theory, which is a commonly used criterion for classification performance measure. We can get a ROC curve by figuring the plotting of the classifier's sensitivity (also known as true positive classification rate) as a function of the classifier's specificity (also known as false positive rate). Sensitivity is a probability of correctly classifying a target object. Specificity is a probability of incorrectly classifying a non-target object. The area under the ROC curve (Az) is defined as an accepted way to compare the classifiers performance. Higher Az would characterize the greater discrimination capacity. A good classifier should have a true positive rate of 1.0 (or 100%) and the false positive rate of 0.0 accordingly with respect to an Az of 1.0.

### 3.2. MCs Classification Based on Sparse Representation

Ideally, the nonzero entries in the estimated **c** will be associated with all the columns in **A** from a single category. So we can easily designate the test image **y** to that category. However, because of the existence of noise, the nonzero entries sometimes may be related to multiple categories. A lot of the-state-of-art classifiers can resolve the problem. For example, we can simply designate **y** to the category with the largest entry of **c**. However, such heuristics cannot model the structure of the subspace associated with MCs blocks.

To better model the structure, we classify **y** based on how much the coefficients relate to the training sample of each category reproducing  **y** alternatively. For each category *i* we define the corresponding characteristic function *δ*_*i*_ : **R** ^*n*^ → **R** ^*n*^ to select the coefficients associated with the *i*th category. If *x* ∈ **R** ^*n*^, *δ*_*i*_(*x*) ∈ **R** ^*n*^ is a new vector whose nonzero entries are the entries in *x* that belongs to the category *i* and whose entries associated with all the other subjects are zero or very close to zero. The classification algorithm can be summarized as follows.

**Algorithm 1 (MCs detection and classification based on sparse representation (MCs-SRC))**

Input: We have a matrix of training images **A** ∈ **R** ^*m*×*n*^ from two categories (MCs or not), a linear transform **D** ∈ **R** ^*d*×*m*^, and an error tolerance *ε*.

(1) Compute features $\tilde{y}=Dy$ and $\tilde{A}=DA$, and normalize $\tilde{y}$ and columns of $\tilde{A}$ to unit length.
(2) Solve the convex optimization problem:
  (16) $$\begin{array}{c} c=\underset{c^{\prime }\in R^{n}}{\min}{||c^{\prime }||}_{1}\;\text{subject}\;\text{to}\;\;y=Ac,\\ J\left(c,\lambda \right)=\underset{c}{\min}\left\{{||Ac-y||}_{2}+\lambda {||c||}_{1}\right\}. \end{array}$$
(3) Compute the residuals $r_{i}\left(y\right)={||\tilde{y}-\tilde{A}c||}_{2}$ for *i* = +1, −1.

Output: *f*(**y**) = arg min_*i*_ *r*_*i*_(**y**).

### 3.3. MCs Classification Approach with Twin Support Machines and Sparse Representation (TWSVMs-SR)

Given a set of mammogram blocks, each block is transformed and represented in terms of the sparse coefficients with respect to the parts from the vocabulary constructed in the image sparse representation and learning stage. Each block is then transformed into a vector and represented as a sparse feature vector based on the vocabulary parts (the negative and positive samples in the sparse learning set). Then we can use the learned sparse coefficients as each block's sparse feature. So we consider the sparse representation approach as a feature extraction method in terms of the coefficient vector, and then we feed it with the state-of-the-art classifier (TWSVMs) to formulate the detection method as a supervised learning approach.

If we have a set of training blocks labeled as positive (MCs) or negative (non-MCs), each image is represented as a feature vector using the method described above. These feature vectors are then fed as inputs to TWSVMs, which learns to classify an image block as a member or not a member of the object category, under some associated confidence. When we get the learned TWSVMs model, we can use the learned classifier as a reliable detector to perform the detection task.

## 4. Results and Discussion

Up till now, we have illustrated our new methods to detect MCs in mammograms. In this section, we will evaluate the performance of our methods by using the real mammogram data obtained from DDSM. In our experiments, we use the training, test, and validation sets, which were randomly selected from the preprocessed image blocks. The blocks included 3000 with true MCs and 3000 with normal tissue. We chose 75% of the blocks for training and 25% for test.

For a given digital mammogram, we formulate the MCs detection and classification approach as the following steps.

**Step 1**

We first preprocess the mammogram to remove the artifacts, suppress inhomogeneity of the image background, and enhance microcalcifications in the breast area.

**Step 2**

At each pixel location of the image, we manually select a small window (**x** = *A*_*m*×*m*_, where *m* = 115) to describe its surrounding image feature.

**Step 3**

Get the sparse representation of each image block by using the proposed methods.

**Step 4**

Apply the proposed MCs detection methods to decide whether **x** belongs to MCs or not.

In our experiments, they are designed to quantitatively verify the performance of sparse representation based methods for MCs detection and classification by using mammograms. To get an accurate performance measure in this study, a stratified 5-fold cross validation method is employed. We also compared our approaches with the state-of-the-art algorithm, SVMs, which have been successfully applied in MCs detection.

All the experiments are performed on a notebook computer with DUO Intel 2.54 G CPU and 4 G memory under Windows 7. MATLAB 2011 is used to implement sparse representation based MCs detection methods. To get the sparse representation of each image block, we employed the *l*_1__*ls* MATLAB package to achieve the optimization. *l*_1__*ls* is a toolbox in MATLAB implementation of the interior-point method for *l*_1_-regularized least squares solution.

Before we performed the MCs detection methods, we first used the *l*_1__*ls* package to learn the sparse transform matrix with the training dataset. The trained sparse transform matrix is evaluated using all the mammograms in the test dataset. Because TWSVMs do not vary significantly over a wide range of parameter settings, we chose the fixed parameters of TWSVMs, having an RBF kernel with *σ* = 15 and *c*_1_ = *c*_2_ = 1000 in our experiments.

**Figure 3:**
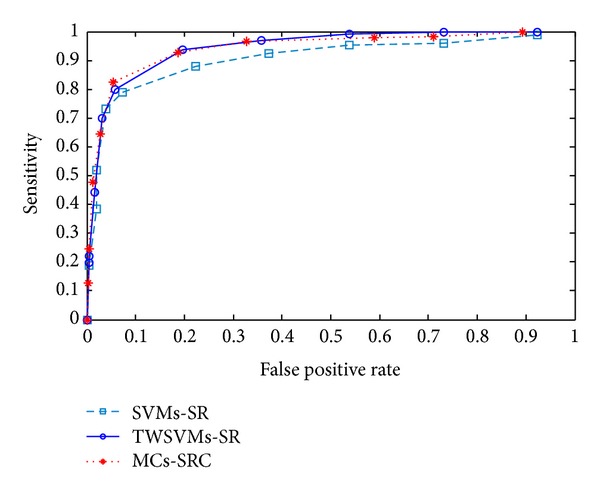
Comparisons of ROC curves of MCs detection and classification using the proposed methods.

First, we did the experiments using MCs-SRC algorithm to perform the MCs detection and classification. The test results are summarized with ROC curves in Figure 3 for the MCs-SRC method. For comparison, ROC curve is also shown for the sparse representation based TWSVMs and SVMs methods with the same inputs.

From Figure 3, it can be shown that the proposed algorithm has a higher detection accuracy rate compared with SVMs and TWSVMs with the same dataset and configuration. By using the same test samples, compared with SVMs and TWSVMs, the proposed method has a better detection performance when we train the classifier. In particular, the proposed method achieved the averaged sensitivity of approximately 92.17% with respect to 7.83% false positive rate and Az = 0.9507. With the same training data set and test data set, the TWSVMs classifier achieved a sensitivity of 90.01%, 9.63% false positive rate, and Az = 0.9459, and SVMs achieved a sensitivity of 87.13%, 9.88% false positive rate, and Az = 0.9298.

To evaluate the stability of our methods, we repeat the sampling 50 times so that we can compute the mean and standard deviation of the detection accuracy, sensitivity, and specificity. We perform the detection and classification task in 20 rounds, and in each round we randomly select training samples from 95% of training samples to 5% to train classifiers. The trained classifiers are evaluated using the other 500 test samples. Average experimental results of the MCs-SRC method, compared with SVMs and TWSVMs, are shown in Table 1.

From Table 1, we can see that the MCs classification with Twin Support Machines and sparse representation (TWSVMs-SR) approach has a bit better performance than the other two. But as we have known, the MCs-SRC method does not need to train a classifier, because the features are acquired by learning, so it will get the classification more efficiently.

## 5. Conclusions

In this paper, a novel approach is described to aid breast cancer detection and classification using digital mammograms. The proposed method is based on sparse feature learning and representation, which expresses a testing sample as a linear combination of the built vocabulary (training samples). The sparse coefficient vector is obtained by using *l*_1_-regularized least square method. MCs detection and classification are achieved by defining discriminating functions from the coefficient vector for each category (called MCs-SRC) and TWSVMs based algorithm using sparse representation as a feature learning method (called TWSVMs-SR). The demonstrated experiments show that TWSVMs-SR method gets the best performance, and MCs-SRC can match the performance achieved by the-state-of-art classifier. Furthermore, the MCs-SRC approach does not need to select optimal model parameters of the used classifier.

## Figures and Tables

**Table 1:** Experimental results of the proposed MCs-SRC method for MCs detection, compared with sparse representation based TWSVMs and SVMs methods.

Methods	Sensitivity	Specificity	Az
MCs-SRC	90.84 ± 1.07%	92.37 ± 0.78%	0.9407 ± 0.0564
TWSVMs-SR	92.07 ± 0.89%	89.93 ± 0.91%	0.9678 ± 0.0977
SVMs-SR	87.53 ± 0.94%	89.72 ± 0.88%	0.9304 ± 0.1001
